# Aberrant antigenic expression in extranodal NK/T-cell lymphoma: a multi-parameter study from Thailand

**DOI:** 10.1186/1746-1596-6-79

**Published:** 2011-08-25

**Authors:** Tawatchai Pongpruttipan, Tanawan Kummalue, Anan Bedavanija, Archrob Khuhapinant, Koichi Ohshima, Fumiko Arakawa, Daisuke Niino, Sanya Sukpanichnant

**Affiliations:** 1 Department of Pathology, Faculty of Medicine Siriraj Hospital, Mahidol University, Thailand; 2 Department of Clinical Pathology, Faculty of Medicine Siriraj Hospital, Mahidol University, Thailand; 3 Department of Otolaryngology, Head and Neck Surgery, Faculty of Medicine Siriraj Hospital, Mahidol University, Thailand; 4 Division of Hematology, Department of Medicine, Faculty of Medicine Siriraj Hospital, Mahidol University, Thailand; 5 Department of Pathology, Kurume University School of Medicine, Japan

**Keywords:** Extranodal NK/T-cell lymphoma, pathology, immunophenotype, EBV, LMP-1 gene, TCR gene rearrangement

## Abstract

**Background:**

Extranodal NK/T-cell lymphoma, nasal type (ENKTL) is not common worldwide, but it is the most common T- and NK-cell lymphomas in many Asian countries. Immunophenotypic profiles were studied based on limited series. The authors, therefore, studied on ENKTL according to characterize immunophenotypic profiles as well as the distribution of EBV subtype and LMP-1 gene deletion.

**Methods:**

By using tissue microarray (TMA), immunohistochemical study and EBV encoded RNA (EBER) in situ hybridization were performed. T-cell receptor (TCR) gene rearrangement, EBV subtyping, and LMP-1 gene deletion were studied on the available cases.

**Results:**

There were 22 cases eligible for TMA. ENKTL were positive for CD3 (91%), CD5 (9%), CD7 (32%), CD4 (14%), CD56 (82%), TIA-1 (100%), granzyme B (95%), perforin (86%), CD45 (83%), CD30 (75%), Oct2 (25%), and IRF4/MUM1 (33%). None of them was positive for βF1, CD8, or CD57. TCR gene rearrangement was negative in all 18 tested cases. EBV was subtype A in all 15 tested cases, with 87% deleted LMP-1 gene. Cases lacking perforin expression demonstrated a significantly poorer survival outcome (p = 0.008).

**Conclusions:**

The present study demonstrated TIA-1 and EBER as the two most sensitive markers. There were a few CD3 and/or CD56 negative cases noted. Interestingly, losses of CD45 and/or CD7 were not uncommon while Oct2 and IRF4/MUM1 could be positive in a subset of cases. Based on the present study in conjunction with the literature review, determination of PCR-based TCR gene rearrangement analysis might not be a useful technique for making diagnosis of ENKTL.

## Background

T-cell lymphoma, especially extranodal NK/T-cell lymphoma, nasal type (ENKTL), has a higher incidence in Asian and Latin American countries than the Western [1-3]. In a recently published series of 71 consecutive mature T- and NK-cell lymphomas in Thailand, ENKTL accounted for 31%, the most common subtype which is higher than other types including anaplastic large cell lymphoma (18%), angioimmunoblastic T-cell lymphoma (14%), peripheral T-cell lymphoma, not otherwise specified (PTCL, NOS, 13%), and other less common subtypes [4].

ENKTL is a type of non-Hodgkin lymphoma, most common in upper aerodigestive tract, particularly nasal cavity [2,3,5]. ENKTL is believed to be derived from either NK- or cytotoxic T-cell, but the former is more common [5,6]. T-cell receptor (TCR) gene rearrangement is mostly in germ line configuration, corresponding to the majority of cases those are of NK-cell lineage [6,7]. Due to the different therapeutic approach and prognostic outcome of ENKTL from other T-cell/NK-cell lymphomas, definite diagnosis for appropriate management is very important [5,8]. While ENKTL needs a different therapeutic approach, but only limited series studied on expanded phenotypic features which important for distinguishing ENKTL from other T-cell lymphomas.

Epstein-Barr virus (EBV) is closely associated with ENKTL [1,2]. It has A or B subtypes as determined by the difference of EBV nuclear antigen 2 (EBNA2) gene sequence [2,9]. And almost all cases of Asian ENKTL are subtype A [2,5,6,10-12], while a Europe, North America and Latin America have variable proportions of subtype B [13-19]. Nevertheless, no EBV subtype has been documented in Thai ENKTL before.

The present study was focused on basic clinical information, histopathology, immunophenotype, and PCR-based TCR gene rearrangement. EBV subtype and EBV LMP-1 gene deletion were also of interest.

## Methods

This study was approved by the Institutional Review Board, Faculty of Medicine Siriraj Hospital, Mahidol University (Si087/2008).

The study samples were newly diagnosed ENKTL during 2004 and 2007 at Department of Pathology, Siriraj Hospital. Known cases of ENKTL, consultation cases from other hospitals without patient visit at Siriraj Hospital, and cases with inadequate material for a making definite diagnosis were excluded.

### Basic clinical features, histopathologic, immunophenotypic, and in situ hybridization studies

All newly diagnosed cases of extranodal lymphoma of either T- or NK type of head and neck region were recruited for review. Clinical information was gathered from those given in the requisitions and medical records. Histopathologic features were reviewed by TP and SS, and partially by DN and KO. The criteria for diagnosis were based on the "WHO Classification of Tumours of Haematopoietic and Lymphoid tissues" published in 2008 [1].

For the cytomorphological aspect, using nuclear size, we divided cases into 6 categories: 1) small, for predominantly small cells (>75% of lymphoma cells); 2) small to medium, for mixture of small to medium-sized cells (at least 25% each); 3) predominantly medium, for predominantly medium-sized cells (>75% of cells); 4) medium to large, for mixture of medium-sized to large cells (at least 25% each); 5) large, for predominantly large cells (>75% of cells); and 6) anaplastic, for a fair amount of anaplastic cell component (at least 25% of cells). In the present study, small cell means cell which had nuclear size similar to a normal lymphocyte; large cell had nuclear size equal or greater than the twice size of a normal lymphocyte; medium-sized cell had nucleus intermediate between small and large cell; and anaplastic cell was large cell with anaplastic features.

A tissue microarray (TMA) block was constructed from formalin-fixed paraffin-embedded (FFPE) tissue (3.5 mm thick cores). The antibodies for immunohistochemical studies included ALK (ALK1, dilution 1:100), BCL2 (124, 1:50), BCL6 (LN22, 1:100), βF1 (8A3, 1:20), CD1a (010, 1:200), CD3 (LN10, 1:600), CD4 (4B12, 1:10), CD5 (4C7, 1:100), CD7 (CBC.37, ready to use), CD8 (C8/144B, ready to use), CD10 (56C6, ready to use), CD15 (MMA, 1:200), CD20 (L26, 1:2,000), CD23 (1B12, 1:100), CD30 (Ber-H2, 1:100), CD34 (M7165, 1:300), CD43 (DF-T1, 1:500), CD45 (LCA, 1:1,500), CD56 (123C3.D5, 1:50), CD57 (TB01, 1:300), CD79a (JCB117, 1:600), CD117 (c-kit, 1:5,000), CD138 (MI15, 1:1,000), cyclinD1 (SP4, 1:200), Interferon regulatory factor-4/Multiple myeloma-1 (IRF4/MUM1) (MUM1p, 1:800), granzyme B (GrB-7, 1:50), BOB.1 (TG14, 1:20), Oct2 (C-20, 1:4,000), perforin (5B10, 1:150), PAX5 (24, ready to use), TIA-1 (TIA-1, 1:500), and TdT (SEN28, 1:100). The immunohistochemical studies and EBV-encoded RNA (EBER) in situ hybridization were performed in an automated staining machine (Benchmark^® ^XT). Criteria for interpretation of immunohistochemistry and EBER in situ hybridization were as follows: 1) mostly positive - definitely positive on lymphoma cells >50%, 2) partially positive -10-50% positive staining, 3) probably negative - <10% staining on presumptive lymphoma cells, and 4) clearly negative -clearly negative. Morphologic and immunophenotypic analyses were based on only cases with adequate tissue in TMA block.

### T-cell receptor gene rearrangement molecular studies

Only cases with adequate tissue on TMA slides were subjected to PCR-based TCR gene rearrangement analysis. Twenty serial 10-μm-thick sections of tissue from FFPE tissue were obtained for molecular genetic studies. The PCR primer sequences were designed following BIOMED-2 protocols [20], which the present study included primers for TCRγ, δ and β genes, and using the previously reported method [21].

### PCR analysis for EBV subtype and LMP-1 deletion

Only cases with adequate tissue on TMA slides were subjected to EBV molecular genetic analysis. After the tissue sectioning for TCR gene rearrangement, the representative tissues from FFPE blocks of each case were sent to study at The Department of Pathology, Kurume University School of Medicine, Japan. The DNA preparation was performed from whole sections of the 22 ENKTL cases. DNA samples were extracted using a commercial kit (Blood & Tissue Genomic Extraction Miniprep System^®^, Viogene). The extracted DNA was used for PCR.

The EBNA 2A and 2B primer sequences for EBV subtyping, previously reported by Ohshima et al [22], were used for DNA amplification (listed in Table 1). The final reaction was incubated at 95°C for 10 min, followed by 50 cycles of 95°C for 30 s, 60°C for 30 s, and 72°C for 30 s. Then, a final extension at 72°C for 10 minutes was performed.

**Table 1:** List of PCR primers used for EBV subtyping and detection of LMP1 gene deletion

Type of Primer			DNA sequence	Product size
EBNA2	EBV type A	2A-5'	5'-AACTTCAACCCACACCATCA-3'	116 bp
	EBV type B	2A-3'	5'-TTCTGGACTATCTGGATCAT-3'	120 bp

EBV LMP-1	wild-type	1-A	5'-CGACTCTGCTGGAAATGATGGAGGC-3'	210 bp
	deletion-type	1-B	5'-TGAACTGGGCCGTGGGGGTCGTCAT-3'	180 bp

β-actin (semi-nested PCR)	1^st ^forward	SFW	5'-CAAGAGATGGCCACGGCTGCT-3'	
	2^nd ^forward	S4FW	5'-GAGGCACTCTTCCAGCCTTCC-3'	148 bp
	reverse	A5RV	5'-TCGTGGATGCCACAGGACTCC-3'	

EBNA2, Epstein-Barr virus nuclear antigen 2 gene; LMP1, latent membrane protein 1 gene

After DNA amplification, the amplified products were subjected to electrophoresis in 3% agarose gel, and were visualized by ethidium bromide staining under ultraviolet light. The expected sizes of EBV type A (EBNA 2A) and B (EBNA 2B) products were 116 and 120 bp in length, respectively.

To test for a 30 bp LMP-1 gene deletion at nucleotide positions of 168, 256-168, and 285, the B95-8 (wild type) EBV gene was used for comparison [23]. Primers (LMP-1-A, LMP-1-B) flanking the characteristic 30 bp deletion were synthesized (Table 1). The condition for DNA amplification and detection of PCR products were the same as for EBV subtyping. The expected PCR products of wild and deletion types were 210 and 180 bp, respectively.

The genomic locus of β-actin was used as an internal control. The semi- nested PCR reaction consisted as follows: after initial denaturation at 95°C for 10 min, 5 cycles of 95°C for 30 s, 63°C for 30 s, and 72°C for 30 s; followed by 45 cycles of 95°C for 30 s, 60°C for 30 s, and 72°C for 30 s, and then with a final extension at 72°C for 10 min. The expected size of genomic β-actin was 148 bp.

### Clinical course

In cases with adequate tissue in TMA, additional clinical data including Ann Arbor stage, international prognostic index (IPI) score, modes of treatment, and survival status was collected. The Kaplan-Meier survival analyses were performed based on various factors.

### Statistical Evaluation

Statistical comparisons between characters were performed using the 2-tailed Fisher exact test (SPSS, v. 18.0, Chicago, IL). Log Rank test (SPSS, v. 18.0) was used for determination of the differences in survival outcome. Statistical significant was defined if p < 0.05.

## Results

There were 31 cases of newly diagnosed ENKTL recruited over the 4-year period. Of the 31 cases, 5 cases of nasal biopsy and 1 case of pharyngeal biopsy had not enough tissue left on paraffin block for further studies. Thus, 25 cases were constructed in a TMA block, but 3 of them were cut through the neoplastic parts which inadequate for interpretation. Therefore, only 22 cases of ENKTL were subjected to immunophenotypic and TCR gene rearrangement studies.

### Basic clinical features

In term of geographic distribution, the patients came from all regions of Thailand, 14 cases (45%) from central region, 11 cases (36%) from Bangkok Metropolitan and periphery, 3 cases (10%) from northeastern region, 2 cases (7%) from southern region, and 1 case (3%) from northern region.

For sites of lymphoma at presentation, 16 cases (52%) had nasal cavity involvement, 1 case (3%) with bone marrow and nasal cavity, 1 case (3%) with nasal cavity and sinuses, 1 case (3%) with nasal and oral cavities, 3 cases (10%) with nasal cavity and nasopharynx, 3 cases (10%) with nasopharynx, 2 cases (7%) with oral cavity (palate and buccal mucosa each), 2 cases (7%) with periorbital tissue, 1 case (3%) with pharynx, and 1 case (3%) with larynx and base of tongue. Among these cases, there were 2 out of 27 patients (7%, case no. 14 and 31) with serologic evidence of HIV infection. Basic clinicopathologic features were shown in Table 2 and the summaries were shown in Table 3.

**Table 2:** Clinical information, morphology, immunophenotype, EBER in situ hybridization, and TCR gene rearrangement of extranodal NK/T-cell lymphoma, nasal type of head and neck region*

No.	Age/Sex	Site (s) of involvement	Symptom (s) and its duration	Basic morphology	CD3	CD5	CD7	CD4	CD8	βF1	CD56	TIA1	GB	PF	CD45	CD30	EBER	TCR gene	Clinical course
1	74/M	Nasal cavity	Rhinorrhea, 1 year	large (A+, N+)	+	-	-	-	-	-	+	+	+	+	+	+	+	I	D, 3 mos
2	40/M	Nasal cavity	Nasal obstruction, 3 mos	medium (A+, N+)	p+	-	-	-	-	-	-	+	p+	p+	-	+	+	I	D, 33 mos
3	31/M	Nasal cavity	Nasal obstruction, 1 mo	large (A+, N+)	p+	-	-	-	-	-	+	+	+	+	+	+	+	-	D, 37 mos
4	37/M	Nasal cavity	Orbital cellulitis, and fever, 2 mos	medium (A-, N+)	p+	-	-	-	-	-	p-	+	p+	+	NA	+	+	-	D, 3 mos
5	41/F	Nasal cavity	Nasal obstruction, 1 mo	small to medium (A+, N+)	+	-	NA	-	-	-	+	+	p+	+	+	NA	+	-	D, 3 mos
6	49/F	Nasal cavity	Nasal obstruction, 2 mos	medium to large (A-, N+)	+	-	+	-	-	-	+	+	+	+	+	p+	+	-	A, 60 mos
7	43/F	Nasal cavity	Nasal obstruction, 2 mos	medium to large (A+, N+)	p+	-	-	p+	-	-	+	+	+	+	-	p+	+	-	D, 8 mos
8	32/M	Nasal cavity	Nasal obstruction, 5 mos	medium to large (A-, N+)	+	-	p-	-	-	-	+	+	p+	p+	NA	-	+	-	D, 3 mos
9	33/M	Nasal cavity	Rhinorrhea, 1 mo	medium (A+, N+)	+	-	+	-	-	-	+	+	p-	p-	p+	+	+	-	D, 4 mos
10	38/M	Nasal cavity	Nasal obstruction with epistaxis, 2 yrs	medium (A+, N+)	+	-	-	-	-	-	p+	+	+	+	+	+	+	I	D, 7 mos
11	48/F	Nasal and oral cavities	Nasal obstruction, 1 mos	medium to large (A+, N+)	+	p+	NA	-	-	-	p+	+	p+	p+	NA	-	+	-	D, 3 mos
12	36/M	Nasal cavity and nasopharynx	Epistaxis with fever, 1 mo	small to medium (A+, N+)	+	-	+	-	-	-	+	+	p+	p-	p+	-	+	-	D, 1 mos
13	26/F	Nasal cavity and nasopharynx	Rhinorrhea, 2 mos	medium (A+, N+)	+	p+	+	-	-	-	+	+	+	+	NA	-	+	-	D, 4 mos
14	42/M	Nasal cavity and nasopharynx	Epistaxis (HIV+), 2 mos	medium to large (A-, N+)	+	-	-	p+	-	-	+	+	+	+	p+	p+	+	-	D, 18 mos
15	41/M	Nasopharynx	Dysphagia, 3 mos	medium (A+, N-)	+	-	-	-	-	-	p+	+	+	+	+	+	+	-	D, 11 mos
16	44/M	Nasopharynx	Rhinorrhea with epistaxis, 6 mos	medium to large (A+, N+)	-	-	-	-	-	-	-	+	p+	-	-	+	+	-	D, 2 mos
17	42/M	Nasopharynx	Nasal obstruction, 1 wk	medium to large (A+, N+)	+	-	+	-	-	-	+	+	+	+	p+	+	+	I	D, 5 mos
18	57/F	Oral cavity	Palatal mass, 2 mos	large (A+, N+)	+	-	-	-	-	-	+	+	+	+	p+	-	+	-	D, 4 mos
19	38/M	Oral cavity	Chronic oral ulcer, 3 mos	medium to large (A-, N+)	+	-	-	-	-	-	-	+	+	p+	+	p+	+	-	D, 3 mos
20	73/M	Base of tongue and larynx	Dysphagia, 2 mos	medium to large (A+, N+)	p+	-	NA	-	-	-	+	+	+	+	p+	NA	+	-	D, 6 mos
21	66/M	Periorbital soft tissue	Orbital cellulitis, 2 wks	medium to large (A+, N+)	-	-	p+	-	-	-	+	+	+	+	p+	+	+	-	D, 3 mos
22	56/M	Periorbital soft tissue	Blurred vision and headache, 2 mos	medium to large (A+, N+)	+	-	-	p+	-	-	+	+	p+	p+	+	p+	+	-	D, 10 mos

* Please see the abbreviations below

For "basic morphology": Large: predominantly (>75%) large cells; Medium to large: mixture of medium-sized to large cells (≥25% each); Medium: predominantly (>75%) medium-sized cells; Small to medium: mixture of small to medium-sized cells (≥25% each); A+: presence of angioinvasion; A-: absence of angioinvasion; N+: presence of tissue necrosis; N-: absence of tissue necrosis

For immunophenotype: GB: granzyme B; PF: perforin; + (mostly positive): positive on lymphoma cells >50%; p+ (partially positive): 10-50% positive; p- (probably negative): <10% staining; - (negative): clearly negative; NA: not available

For TCR gene (PCR -based TCR gene rearrangement analysis); - (negative): no clonal band on polyacrylamide gel electrophoresis and no dominant peak on fluorescence capillary electrophoresis; I: inadequate DNA quality for PCR

For clinical course: A: alive; D: death

**Table 3:** Summary of basic clinico-pathological features of extranodal NK/T-cell lymphoma, nasal type of head and neck region

**Demographic data **(From 31 cases)	Frequency	%
**Age**		
- Range: 15-74 years		
- Median: 42 years		
- Mean: 45 years (SD 13)		
**Sex**		
- Male	23	74
- Female	8	26
- M/F ratio: 2.9		

Major presenting clinical symptoms* (From 31 cases)	**Frequency**	**%**

Nasal obstruction	15	48
Rhinorrhea	4	13
Epistaxis	4	13
Fever	3	10
Dysphagia	3	10
Orbital swelling and pain	2	7
Blurred vision and headache	1	3
Epiphora	1	3
Chronic oral ulcer	1	3
Palatal mass	1	3

**Morphologic patterns **(From 22 cases)	**Frequency**	**%**

**Lymphoma cell size**		
- Predominantly small	0	0
- Mixed small and medium-sized	2	9
- Predominantly medium-sized	6	27
- Mixed medium-sized and large	11	50
- Predominantly large	3	17
- Anaplastic	0	0
**Angioinvasion**		
- Present	15	68
- Not identified	7	32
**Tissue necrosis**		
- Present	21	95
- Not identified	1	5

* Some cases have more than one major presenting symptom

### Histopathologic, immunophenotypic, EBER in situ hybridization, and T-cell receptor gene rearrangement molecular studies

Results of immunophenotype, EBER in situ hybridization and TCR gene rearrangement of the studied cases were shown in Table 2. The summary of immunophenotypic features were demonstrated in Table 4. In addition to the results in Table 4, all tested cases were negative for CD1a (19 cases), CD10 (17 cases), CD15 (21 cases), CD57 (20 cases), CD23 (16 cases), CD34 (22 cases), CD79a (18 cases), CD117 (16 cases), CD138 (18 cases), TdT (18 cases), cyclin D1 (22 cases), PAX5 (22 cases), BOB.1 (17 cases), BCL2 (18 cases), and BCL6 (18 cases).

**Table 4:** Summary of immunohistochemical results of the 22 cases of extranodal NK/T-cell lymphoma, nasal type

Marker	Total cases	Mostly positive	Partially positive	Overall positivity	Probably negative	Clearly negative	Overall negativity
**CD3**	**22**	15 (68%)	5 (23%)	**20 (91**%)	0	2 (9%)	**2 (9**%)
**CD20**	**22**	0	0	**0**	0	22 (100%)	**22 (100**%)
**CD5**	**22**	0	2 (9%)	**2 (9**%)	0	20 (91%)	**20 (91**%)
**CD7**	**19**	5 (26%)	1 (5%)	**6 (32**%)	1 (5%)	12 (63%)	**13 (68**%)
**CD4**	**22**	0	3 (14%)	**3 (14**%)	0	19 (86%)	**19 (86**%)
**CD8**	**22**	0	0	**0**	0	22 (100%)	**22 (100**%)
**βF1**	**22**	0	0	**0**	0	22 (100%)	**22 (100**%)
**CD56**	**22**	15 (68%)	3 (14%)	**18 (82**%)	1 (5%)	3 (14%)	**4 (18**%)
**CD57**	**21**	0	0	**0**	0	21 (100%)	**21 (100**%)
**TIA-1**	**22**	22 (100%)	0	**22 (100**%)	0	0	**0**
**Granzyme B**	**22**	13 (59%)	8 (36%)	**21 (95**%)	1 (5%)	0	**1 (5**%)
**Perforin**	**22**	14 (64%)	5 (23%)	**19 (86**%)	2 (9%)	1 (5%)	**3 (14**%)
**CD45**	**18**	8 (44%)	7 (39%)	**15 (83**%)	0	3 (17%)	**3 (17**%)
**CD43**	**22**	20 (91%)	2 (9%)	**22 (100**%)	0	0	**0**
**CD30**	**20**	10 (50%)	5 (25%)	**15 (75**%)	0	5 (25%)	**5 (25**%)
**ALK**	**18**	0	0	**0**	0	18 (100%)	**18 (100**%)
**OCT2**	**20**	1 (5%)	4 (20%)	**5 (25**%)	3 (15%)	12 (60%)	**15 (75**%)
**IRF4/MUM1**	**15**	1 (7%)	4 (27%)	**5 (33**%)	0	10 (67%)	**10 (67**%)

Mostly positive = definitely positive on lymphoma cells >50%

Partially positive = 10-50% positive staining

Overall positive = sum of mostly and partially positive results

Probably negative = <10% staining on presumptive lymphoma cells

Clearly negative = clearly negative

Overall negative = sum of probably negative and clearly negative results

### PCR analysis for EBV subtype and LMP-1 deletion

After DNA extraction from FFPE tissue and DNA amplification, 18 of 22 cases (82%) had adequate DNA to perform TCR gene rearrangement analysis. All tested cases showed negative results on both gel and fluorescence capillary electrophoresis (see Table 2).

For EBV subtyping and LMP-1 gene deletion, 15 of 22 cases had adequate tissue for these studies. All of the15 cases were determined as EBV subtype A and 13 of 15 cases (87%) had LMP-1 gene deletion type, while the other 2 cases (13%) had wild type.

### Clinical course

Of the 22 cases, patients with stage I, II and IV, were 13 (62%), 1 (5%) and 7 cases (33%), respectively. Among 29 cases, there were 2 cases (7%) with bone marrow involvement at the time of diagnosis. Of the 18 cases with available information, patients with IPI score of 0, 1, 2, 3 and 4, were 7 (39%), 3 (17%), 3 (17%), 3 (17%) and 2 cases (11%). Nineteen of the 22 cases (86%) were treated with only chemotherapy, while 3 cases (no. 2, 3 and 8) were treated with combined chemoradiotherapy.

Of the 22 cases, all patients but one died with a median survival of 4 months. The only survived patient who had stage I disease and received only chemotherapy had been followed up for 5 years. Adverse prognostic factors included high Ann Arbor stage (stage IV vs. stage I or II, survival of 3 vs. 7 months, p = 0.015), higher IPI score (IPI score ≥1 vs. 0, 3 vs. 10 months, p = 0.003), and lack of perforin expression (without vs. with perforin expression, 2 vs. 4 months, p = 0.008). While the expression of other markers including CD45, CD30, CD7, Oct2 and IRF4/MUM1 failed to show any significant difference in survival outcome.

## Discussion

ENKTL has a significant higher incidence in Asian and Latin American countries than the Western [1,2,24]. From the published series of 1983 cases of malignant lymphoma from Thailand, T-cell lymphoma accounted for 23% among overall new lymphoma cases [25], but the frequency of ENKTL was not reported due to the lack of markers for making a definite diagnosis. However, there were 69 cases of the so-called "angiocentric lymphoma" in the REAL classification, a predecessor of ENKTL, among the 381 cases of mature T-cell lymphoma in the series, thus the frequency of angiocentric lymphoma was approximately 18% of mature T-cell lymphoma, the second most common mature T- and NK-cell lymphoma after PTCL, NOS, according to the previous version of WHO classification (2001) [25]. A recently published article from Thailand showed ENKTL as the most common among the mature T- and NK-cell lymphomas [4], accounting for 31%, which is comparable to Taiwan (26%), Korea (37%) and Hong Kong (39%) [26-28], but it is more common when compared to China (up to 4.7%) [29], Japan (6%) [30] and India (8%) [31]. The difference of ENKTL proportion between Thailand and India may be due to the difference in the ethnic origin. In most parts of Japan, proportion of ENKTL is not high, but it is the second most common mature T- and NK-cell lymphoma in Okinawa; and if exclude cases of adult T-cell lymphoma/leukemia that is ultimately higher than other countries, ENKTL proportion in this region will be 34% and seems to be comparable to Thailand, Taiwan, Korea and Hongkong [30]. The reasons for a lower ENKTL proportion in most parts of Japan and mainland China are not known.

In the study period of the present study, besides the 31 ENKTL cases, there was only one extranodal EBV-negative T-cell lymphoma identified from head and neck region. It was a PTCL, NOS, involving nasopharynx, and was positive for CD3, CD4, CD5, TIA-1, βF1, but negative for CD8, CD20, CD56, CD30 and ALK. Unfortunately, TCR gene rearrangement could not be testified in this case due to the inadequate extracted DNA.

ENKTL in the present study showed a strong male predilection with male to female ratio of 2.9, similar to the other previous reports [5,24]. In the present study, the most common presenting symptom was nasal obstruction, similar to the other previous reports [5,24]. The most common site of involvement was nasal cavity while the second most common site was nasopharynx. Nearly 84% of the cases (26/31) involved nasal cavity and/or nasopharynx. In Thailand, a previous study showed that ENKTL was the most common lymphoma in nasal and paranasal sinuses while it might be less common than diffuse large B-cell lymphoma in nasopharynx [32]. In the present study, 2 of 31 cases (7%) had periorbital soft tissue involvement; both cases showed periorbital soft tissue involvement together with mild haziness of paranasal sinuses demonstrated by CT scan, while no lesion was observed from endoscopic examination. Unfortunately, no pathologic sample was sent for proving the occurrence of nasal disease. Thus, it is not possible to conclude whether these 2 cases had concomitant paranasal sinus involvement or they were a genuine primary orbital lesion which was rarely reported [33].

ENKTL with bone marrow involvement at presentation is rare. There were 2 out of 29 cases (7%) in the present study, similar to other series [16,24,34,35]. In particular cases with obvious marrow involvement, distinction between an aggressive NK-cell leukemia and a leukemic phase of ENKTL should be problematic [1]. A presence of nasal or nasopharygeal lymphoma would be favored for ENKTL.

The present study demonstrated a high proportion of cases with necrosis, similar to most other studies, while evidence of angioinvasion varied [16,34,36]. The varied proportion of necrosis among these studies presumably depends on tissue sampling whether necrotic area was included in a small biopsy or not. For the varied proportion of angioinvasion, it could be resulted from either a subjective determination by pathologists or from the amount of tissue obtained for evaluation.

Cytological aspects of ENKTL varied from series to series. In the present study, most cases (77%, 17/22 cases) had predominantly medium-sized cells or mixed medium-sized and large cells. The lymphoma with medium-sized cells commonly showed oval irregular nuclei with occasional nuclear elongation, inconspicuous nucleoli. Cases with large number of small cells might be difficult to distinguish from reactive processes, as a case found in the present study had been misdiagnosed as fungal infection in the first biopsy. In this situation, even presence of necrosis and fungi in the nasal cavity, making diagnosis of fungal infection without careful evaluation of the lymphoid component was dangerous. Thus, careful evaluation of lymphoid cells along with immunohistochemistry and EBER in situ hybridization might be helpful for making a diagnosis of lymphoma. In our experience, a few cases showing monotonous small lymphoma cells or anaplastic morphology were also observed, but they were not in the study period; such cases are uncommon as described in the WHO classification blue book [5].

An advantage of TMA technique, other than cost and time effectiveness, is that the tissue samples can act as multiple controls within a single slide. The relatively small tissue did not affect the interpretation much, since most samples were from small cup biopsies. According to the TMA technique, most of the cases in the present study had typical ENKTL immunophenotype but with minor differences to other series. The details were discussed below.

For the T-cell markers, CD3 is a highly sensitive marker and helpful for diagnosing ENKTL. However, CD3-negative ENKTL were account for 9% (2/22 cases) in the present study, which were also previously documented from other series [6,15,37]. Thus, negative for CD3 does not rule out ENKTL. For other T-cell markers, CD5 is negative in almost all ENKTL, similar to previous study [5]. The present study had only 2 partially CD5-positive cases. This could be resulted from CD5 on reactive T-cells which sometime difficult to distinguished from neoplastic cells. Since there was no any case with expression of β TCR protein as well as the absence of demonstrable TCR gene rearrangement, these results probably reflect that at least almost all cases were genuine NK-cell in origin. However, we did not have antibodies react with γ or δ TCR to determine whether they were γδ T-cell derived ENKTL which they might have similar basic immunophenotype to that of the NK-cell derived ENKTL. In contrast to CD5, CD7 is normally expressed by normal NK-cells, but the present study demonstrated that most of ENKTL (68%) lack CD7 expression, which is similar to the previous studies [29,38]. For CD43, even though it is a highly sensitive marker for ENKTL, as demonstrated in the present study as well as in other series [7,18], it is less useful for subclassifying of T- and NK-cell neoplasms. For CD4, we found a few cases (14%) with some staining as designated as partially positive. However, in these cases, distinction from CD4 expression by the admixed reactive histiocytic and dendritic cells might be difficult. For CD8, there was no positive case in the present series. However, a previous study demonstrated up to 22% (9/41 cases) of CD8-positive ENKTL which those cases were also positive for CD56, but only 2 of 8 cases had clonally rearranged PCR-based TCRγ gene [36].

CD56 is a very helpful marker for diagnosing of ENKTL, especially for distinguishing from reactive processes which usually have only rare or a few scattered CD56-positive small cells. The present study demonstrated a high sensitivity of CD56 immunohistochemical staining as 82% (18/22 cases), comparable to some previous studies such as 74% (31/42 cases) by Ng et al and 82% (37/45 cases) by Ko et al [34,36]. Higher CD56 sensitivity has also been reported such as 97% (36/37 cases) by Barrionuevo et al [16] and 100% (22/22 cases) by Kuo et al [35]. Interestingly, a study using flow cytometric immunophenotyping demonstrated all ENKTL cases expressed CD56 [39]. Furthermore, a study on frozen section immunohistochemistry also demonstrated all cases of ENKTL had CD56 expression [40]. Hypothetically, the variation in detection of CD56 expression possibly reflects false negative results from immunohistochemical technique performed on FFPE tissue, since this technique might not be able to detect the cases with weak CD56 expression. In addition, durability of CD56 antigen in tissue, sensitivity of antibody clones, and staining techniques should be considered to improve the immunophenotypic results.

For the cytotoxic granules, as shown in Table 2 and 4, TIA-1 is the most sensitive cytotoxic protein in ENKTL, which some other studies also demonstrated 100% sensitivity of TIA-1 expression [18,35,36,41]. On the other hand, some studies showed the lacking of TIA-1 expression in a few cases [34,37]. In some series, granzyme B was expressed in all cases tested [18,42]. In addition to the highest sensitivity of TIA-1, it also was the most intense staining when compared to granzyme B and especially to perforin, based on the present study. Perforin is the least sensitive cytotoxic marker has also been reported [18]. In term of lymphoid biology, since both granzyme B and CD30 were considered as activated-cell markers [43,44], and they were found to be positive in most cases. These results support an activated NK-cell phenotype of ENKTL, as it is believed. Moreover, a recent study on gene expression profiling also demonstrated that ENKTL appeared to have a similar expression profile to activated NK-cell rather than a normal non-activated one [45].

CD30 was positive in 75% (15/20 cases) of cases in the present study, higher than other previous studies such as 48% (13/27) by Ko et al and 41% (9/22) by Kuo et al [34,35]. When compared to the CD30-negative cases, there was no significant correlation of CD30 with other immunostaining, patient's age or site of involvement. Cautiously, it is a potential difficulty for distinguishing between CD56-, CD30+ ENKTL and CD56+ anaplastic large cell lymphoma (ALCL), since 15% of ALCLs can be positive for CD56 [46]. In this situation, presence or absence of EBV or ALK protein may be helpful for making a definite diagnosis.

CD45 (leukocyte common antigen) was not expressed by some ENKTL in the present series. And this should not be confused with a non-hematologic malignancy. To the best of our knowledge, loss of this marker on paraffin-section immunohistochemistry has never been reported.

For B-cell markers, CD20, CD79a, PAX5 and BOB.1 were generally negative. Interestingly, some cases were positive for Oct2, most of them showed focal and weak positivity, however, a case with diffusely- and strongly-positive lymphoma cells was documented. The expression of Oct2 in 1/2 ENKTL as well as some other types of T-cell lymphomas was previously reported by Saez et al [47]. But its significance is not known. Therefore, study in more cases as well as in biologic detail might be of interest.

IRF4/MUM1 is normally expressed in melonocytes, plasma cells, B-cells and activated T-cells [48]. Besides its expression in B-cell neoplasms, a variable IRF4/MUM1 expression in systemic and cutaneous T-cell lymphoproliferative disorders was reported [49]. The IRF4/MUM1 expression has also been reported in 1 of 3 ENKTL by Natkunam et al [50]. While more recent studies from other regions of the world did not evaluate IRF4/MUM1 expression in their case series [3,15]. The significance of IRF4/MUM1 expression is not known, and might be considered for further study in the future.

Of the 18 cases with PCR-based TCR gene rearrangement study, which using BIOMED-2 primer design, none of them showed positive result from both gel and capillary fluorescence electrophoresis, which similar to some previous studies [37,51,52]. On the other hand, some studies using PCR-based techniques, demonstrated TCR-gene rearranged ENKTL in a variable proportion, such as 8% (1/12 cases) by Gaal et al [18], 9% (7/74) by Gualco et al [15], 10% (3/31) by Ko et al [34], 27% (11/41) by Ng et al [36], 30% (3/10) by Lin et al [53], and up to 71% (10/14) by Mitarnun et al [54]. The varied results of PCR-based TCR gene rearrangement among these studies could be caused by the differences in techniques and designed primers. While using Southern blot analysis, the gold standard for TCR gene rearrangement analysis, Suzumiya et al demonstrated rearrangement only in 8% (1/13) of cases, but with the same technique, Nakamura et al failed to demonstrate rearrangement in all their 6 cases [6,7].

For the specificity of PCR-based TCR gene rearrangement, it is noteworthy to emphasize that positive results can also be found in a significant proportion of B-cell lymphomas [55,56], acute non-lymphoid leukemia [57], and even in reactive lymphoid tissues [58]. Thus, cases with positive PCR-based TCR gene rearrangement should not be always indicated as a T-cell lineage.

Interestingly, a study revealed genuine γδ T-cell lines of nasal ENKTL, verified by demonstration of surface γδ TCR by flow cytometry and immunophenotypic studies, and was also positive for TCR gene rearrangement [59]. Such cases may be responsible for some ENKTL with rearranged TCR gene, together with a few cases of commonly known αβ-TCR-positive ENKTL. However, methods for γ- or δ- TCR staining for demonstration of γδ T-cell origin were not available in the present study.

EBV subtype A was noted from all tested cases (15 cases). As far as we know, the present study is the first to demonstrate EBV subtype of ENKTL in Thailand. The high prevalence of this subtype is similar to those reports from East and Southeast Asia [2,5,6,10-12]. In the non-Asian countries, while subtype B was found in the majority of ENKTL in Peru, USA, and Germany [13,16,18], but subtype A was predominant in Mexico, Chile, Brazil, and Spain [14,15,17,19]. Furthermore, Gualco et al recently described the striking differences of EBV subtype among geographic regions in Brazil which may reflect the heterogeneity of the ancestral population [15].

In the present study, 87% of cases were found to be the deletion type of EBV LMP-1 genes, similar to other studies [5,6,12,35]. Since LMP-1 protein on EBV-positive lymphoid cells is an antigenic target for cytotoxic T-cells, high frequency of the deletion-type in ENKTL compared to reactive conditions which wild-type are more common, might suggest a clonal selection of immunologically escapable EBV-infected cells in neoplastic processes [12].

The present study also demonstrated a highly aggressive clinical course of ENKTL which was widely known. Upfront radiotherapy was proved by recently published studies to improve the survival outcome [5,8]. However, most of the patients in this series received only chemotherapy, and there were too few patients received combined chemo-radiation therapy to do a statistic comparison with.

The present study demonstrated a significant poor survival outcome in cases without perforin expression. The cause of aggressive behavior is not known, but loss of perforin expression possibly reflects the more complex genetic abnormality in the tumor cells.

## Conclusions

In conclusion, ENKTL is a distinctive T-/NK-cell lymphoma characterized by tissue necrosis, frequent angioinvasion, EBV association, expression of cytotoxic molecule (TIA-1), cytoplasmic CD3, and CD56 but lacking CD5. Interestingly, losses of CD45 and CD7 were not uncommon. Furthermore, Oct2 and IRF4/MUM1 expression could also be found in ENKTL. As ENKTL needs a different therapeutic approach, any abnormal lymphoid proliferation at upper aerodigestive tract should be highly concerned for ENKTL. The diagnosis should be relied on morphology, immunophenotype and EBER in situ hybridization. However, based on the present study conjunction with literature review, determination of TCR gene rearrangement by PCR-based analysis may not be useful for making diagnosis of ENKTL.

## Competing interests

The authors declare that they have no competing interests.

## Authors' contributions

TP, SS and KO participated in the design of the study. TP, SS, ND and KO reviewed H&E slides. TP constructed TMA block. TP and SS reviewed immunohistochemical slides. FA carried out the EBV subtyping and LMP-1 gene deletion analysis. TK carried out the molecular genetic studies for TCR gene rearrangement analysis. TP, AK and AB collected the clinical data. TP performed the statistical analysis and wrote the manuscript. All authors read and approved the final manuscript.
